# Biomarker Selection and Classification of “-*Omics*” Data Using a Two-Step Bayes Classification Framework

**DOI:** 10.1155/2013/148014

**Published:** 2013-09-11

**Authors:** Anunchai Assawamakin, Supakit Prueksaaroon, Supasak Kulawonganunchai, Philip James Shaw, Vara Varavithya, Taneth Ruangrajitpakorn, Sissades Tongsima

**Affiliations:** ^1^Department of Pharmacology, Faculty of Pharmacy, Mahidol University, 447 Sri-Ayuthaya Road, Rajathevi, Bangkok 10400, Thailand; ^2^Department of Electrical and Computer Engineering, Faculty of Engineering, Thammasat University, 99 Phahonyothin Road, Khlong Nueng, Khlong Luang, Pathum Thani 12120, Thailand; ^3^National Center for Genetic Engineering and Biotechnology, 113 Thailand Science Park, Phahonyothin Road, Khlong Nueng, Khlong Luang, Pathum Thani 12120, Thailand; ^4^Department of Electrical and Computer Engineering, King Mongkut University of Technology North Bangkok, 1518 Piboonsongkarm Road, Bangkok 10800, Thailand; ^5^Language and Semantic Technology Laboratory, National Electronic and Computer Technology Center, 112 Thailand Science Park, Phahonyothin Road, Khlong Nueng, Khlong Luang, Pathum Thani 12120, Thailand

## Abstract

Identification of suitable biomarkers for accurate prediction of phenotypic outcomes is a goal for personalized medicine. However, current machine learning approaches are either too complex or perform poorly. Here, a novel two-step machine-learning framework is presented to address this need. First, a Naïve Bayes estimator is used to rank features from which the top-ranked will most likely contain the most informative features for prediction of the underlying biological classes. The top-ranked features are then used in a Hidden Naïve Bayes classifier to construct a classification prediction model from these filtered attributes. In order to obtain the minimum set of the most informative biomarkers, the bottom-ranked features are successively removed from the Naïve Bayes-filtered feature list one at a time, and the classification accuracy of the Hidden Naïve Bayes classifier is checked for each pruned feature set. The performance of the proposed two-step Bayes classification framework was tested on different types of -*omics* datasets including gene expression microarray, single nucleotide polymorphism microarray (SNParray), and surface-enhanced laser desorption/ionization time-of-flight (SELDI-TOF) proteomic data. The proposed two-step Bayes classification framework was equal to and, in some cases, outperformed other classification methods in terms of prediction accuracy, minimum number of classification markers, and computational time.

## 1. Introduction

In recent years, the advent of technologies such as microarrays, proteomics, and next-generation sequencing has transformed life science. The data from these experimental approaches provide a comprehensive picture of the complexity of biological systems at different levels. Within each of these “*-omics*” data strata, there exists a small amount of information relevant to particular biological questions, for example, indicative markers or biomarkers (for short) that can accurately predict (classify) phenotypic outcomes. Various machine learning techniques have been proposed to identify biomarkers that can accurately predict phenotypic classes by learning the cryptic pattern from -*omics* data [1]. There are three main categories of machine learning methods for biomarker selection and phenotypic classification, namely, *filter*, *wrapper*, and *embedded* [2]. These methods differ in the degree of computational complexity and prediction accuracy outcomes.

Filtering methods are the least computationally complex and are used to identify a subset of the most informative features from *-omics* data to assist the following classification process. These approaches operate by generating a value for each marker according to their degree of correlation with a given phenotype (class label), and then markers are ranked. However, filter methods are subject to selection of redundant biomarkers; furthermore, these methods cannot explore solutions that require more than one marker to predict the underlying classes. A common filter method is the well-known Student's *t*-test, which is popular because of its simplicity [7].

Wrapper methods iteratively perform combinatorial biomarker search aiming to optimize the predictive power of a classification model. Since this combinatorial optimization process is computationally complex, NP-hard problem, many heuristic have been proposed, for example, [8], to reduce the search space and thus reduce the computational burden of the biomarker selection. 

Similar to wrapper methods, embedded methods attempt to perform feature selection and classification simultaneously. Embedded methods, however, integrate feature selection into the construction of classification models. Recursive feature elimination support vector machine (SVM-RFE) is a widely used technique for analysis of microarray data [9, 10]. The SVM-RFE procedure constructs a classification model using all available features, and the least informative features for that particular model are eliminated. The process of classification model building and feature elimination is repeated until a model using the predetermined minimum number of features is obtained. This approach is thus computationally impractical when a large number of features are considered, since many iterations of the algorithm are required.

Another approach for performing class prediction is Naïve Bayes (NB). The NB learning model relies on Bayes probability theory, in which attributes are used to build a statistical estimator for predicting classes. NB is the simplest form of the general Bayesian network in which all attributes are assumed to be independent. This assumption is not valid for biological systems, in which complex networks of interactions exist, that is, gene regulation; hence, NB has not received much attention for predicting biological classes. Nevertheless, modified Bayesian classification approaches which account for dependencies among features can accurately predict biological classes. Notable examples include selective Bayesian classifiers (SCB) [11], tree-augmented Naïve Bayes (TAN), and averaged one-dependence estimators (AODE) [12]. The Hidden Naïve Bayes (HNB) classifier approach has recently been claimed to show significant improvement over other NB techniques [13]. HNB uses a discrete structural model and hence requires the discretization for preprocessing with continuous signal attributes, for example, expression microarray data.

In this paper, a hybrid statistic-based machine learning approach is suggested that utilizes a two-step heuristic to dramatically reduce the computational time required by HNB, while maintaining high-prediction accuracy when comparing with the other state-of-the-art machine learning techniques. Our proposed two-step framework includes (1) attribute filtering using Naïve Bayes (NB) to extract the most informative features and thus greatly reduce the number of data dimensions and (2) the subsequent higher order classification using Hidden Naïve Bayes (HNB). HNB can be used to construct a high-dimensional classification model that takes into account dependencies among the attributes for analysis of complex biological -omics datasets containing dependencies of features. The performance of the proposed two-step Bayes classification framework was evaluated using datasets from SNParray, cDNA expression microarray, and SELDI-TOF proteomics. The proposed framework was compared with SVM-RFE in terms of classification accuracy, area under the ROC curve (AUC), sensitivity, specificity, and the number of informative biomarkers used for classification. 

## 2. Results and Discussion

In order to understand how a two-step Bayes classification framework can be used to analyze -omics data, the experiments in this section were performed in three different scenarios. First, we need to know if Naïve Bayes (NB) filtering can select good (highly informative) candidate biomarkers, for example, SNPs, genes, or proteins for construction of an accurate classification model. Secondly, we need to demonstrate that the two-step Bayes classification framework is at least as good as a state-of-the-art method such as SVM-RFE. Standard performance metrics were used to carry out the head-to-head comparison. Finally, we show how the two-step Bayes classification framework can also be applied to other kinds of -omics datasets, in which SNP genotyping dataset and proteomic profiles from SELDI-TOF were analyzed. 

### 2.1. Evaluation of Naïve Bayes Filtering

**Figure 1:**
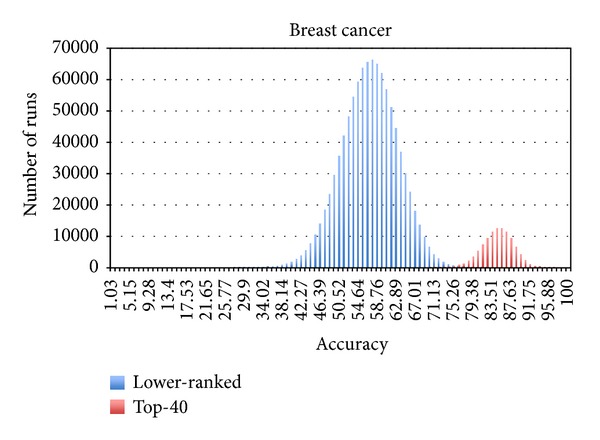
Empirical testing of NB selection using breast cancer dataset. Training breast cancer dataset was sampled 1 million times for lower-ranked marker set and 100,000 times for the top 40-ranked marker set.

**Figure 2:**
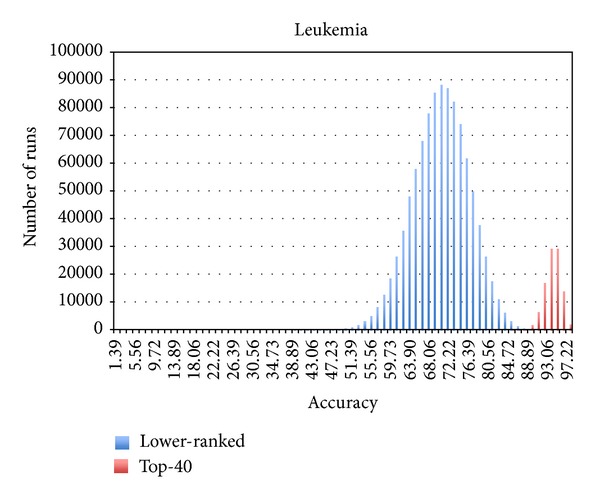
Empirical testing of NB selection using leukemia dataset. Training leukemia dataset was sampled 1 million times for lower-ranked marker set and 100,000 times for the top 40-ranked marker set.

**Figure 3:**
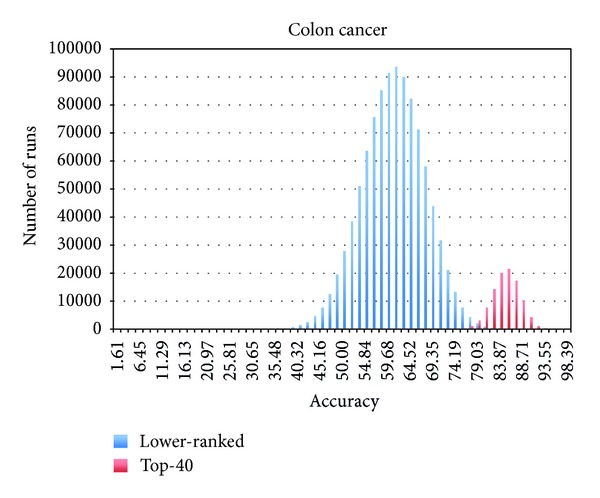
Empirical testing of NB selection using colon cancer dataset. Training colon cancer dataset was sampled 1 million times for lower-ranked marker set and 100,000 times for the top 40-ranked marker set.

First, we hypothesized that the Naïve Bayes (NB) ranking module can precisely extract the most informative biomarkers to maximize the accuracy of the corresponding classification model. To our knowledge, the use of NB as a filter method for identifying highly informative markers is novel. NB allows us to interrogate each marker separately if it can predict the class outcomes with high confidence. The marker can be combined with other informative markers and collectively improve the prediction accuracy in successive multifeature classification HNB step. The experiments were performed using three microarray datasets, namely, breast cancer (24481 genes), leukemia (7129 genes), and colon cancer (2000 genes), from the Kent Ridge Biomedical Data Set Repository (KRBDSR) [3]. The NB and HNB modules from the popular open source machine learning software, Waikato Environment for Knowledge Analysis (Weka) [14], were employed for the two-step Bayes classification framework. The NB module was used to select the top features (genes), whose prediction accuracies are greater than or equal to 75%. Using this criterion, approximately 40 genes were selected by the NB filtering module as the top-ranked informative markers. From empirical testing of several datasets, we have found that this filtering criterion is broadly applicable for reducing the number of markers to a level practical for the subsequent HNB module, without reducing the accuracy of the final HNB classification. The *sampling-with-replacement* of 20 markers was done from both the top 40 group as well as the remaining unselected markers in the three datasets. The classification accuracy of each sampling was tested using the Hidden Naïve Bayes (HNB) module with 10-fold cross-validation classification available in Weka. Twenty genes were sampled from the selected top 40 and the unselected lower-ranked genes for 100,000 and 1 million times, respectively. The frequencies for each classification accuracy event were recorded. The results for the breast cancer, leukemia, and colon cancer data are shown in Figures 1, 2, and 3, respectively. Most importantly, sampling from the top 40 NB-selected genes gives the highest prediction accuracy, and the density distribution plots from the selected top 40 and unselected lower-ranked genes give minimal or no overlap. These results suggest that the NB filtering module is effective for selection of the most informative markers to be used in the following classification model construction by HNB. The threshold of top-ranked m-genes could be optimized for each type of dataset; that is, more or fewer than 40 markers may give slightly better prediction accuracy in the final HNB constructed model. However, in this paper, we did not exhaustively test different m-thresholds, as our focus is more to demonstrate the NB-HNB combination approach.

When the top NB selected genes were used for classification by HNB, the prediction accuracy was excellent for the leukemia dataset (average accuracy 92.90%; range 100% to 87.5%) and good for the breast (average 84.67%; range 96.90–70.10%) and colon cancer datasets (average 86.53%; range 96.77–70.97%). In contrast, the HNB prediction accuracy using markers from the lower-ranked unselected genes was markedly poor: breast cancer average prediction accuracy 57.16% (range 84.54–27.84%), leukemia average accuracy 72.14% (range 97.22–40.28%), and colon cancer average accuracy 50.16% (range 53.16–30.65%). 

It should be noted that NB filtering is not a good realistic statistical model because of the underlying independency assumption among the features (see Section 4.2). In other words, the top NB selected attributes may not always contain the optimal set of features for classification. Nonetheless, when feeding the NB top-ranked attributes to the successive HNB step, HNB was able to better construct a higher order interaction prediction model from these features without exhaustively searching for all different combinations.

### 2.2. Head-to-Head Comparison with SVM-RFE

In order to clearly demonstrate the performance of the two-step Bayes classification framework, a head-to-head performance evaluation between the state-of-the-art machine learning technique, recursive feature elimination support vector machine (SVM-RFE), and our proposed framework was performed. There are 42 previously published SVM-RFE analyses for comparison (see full listing in Section 4). The performance of the two-step Bayes classification framework was compared with the results published in [15]. Nine different machine learning techniques, grouped as filtering, wrapper, and hybrid methods, were compared using breast cancer, leukemia, and colon cancer datasets from KRBDSR. The criteria used to measure the performance of different methods include prediction accuracy, sensitivity, specificity, and the number of selected genes. We tested the proposed two-step Bayes classification framework against these datasets and augmented our performance in conjunction with the tables published in [15]. Tables 1, 2, and 3 show the results from our proposed framework (NB-HNB) in comparison with other methods. NB-HNB outperformed other machine learning methods in terms of prediction accuracy, sensitivity, and specificity. The greater marker requirement of NB-HNB indicates that the Naïve Bayes filtering probably did not rank the top dependent features that can optimally construct an accurate classification model in the correct order. Hence to achieve 100% accuracy from the training set, HNB required more genes to classify.

**Figure 4:**
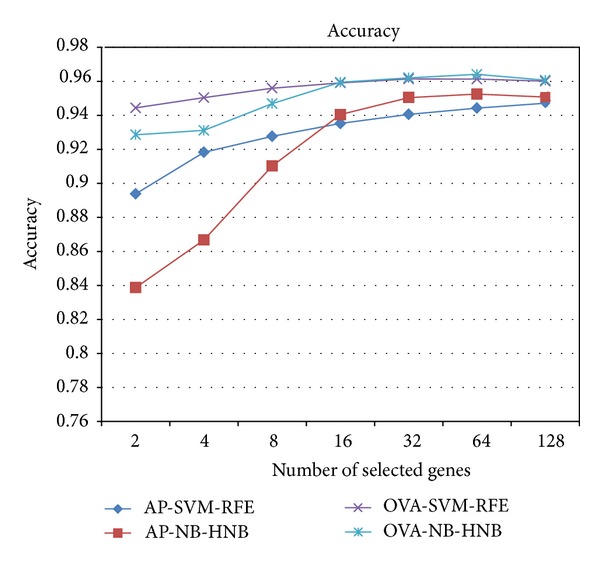
Comparison of average accuracy results over all datasets (Avg), 35 All-Paired datasets (AP) and 9 One-Versus-All (OVA) datasets.

**Figure 5:**
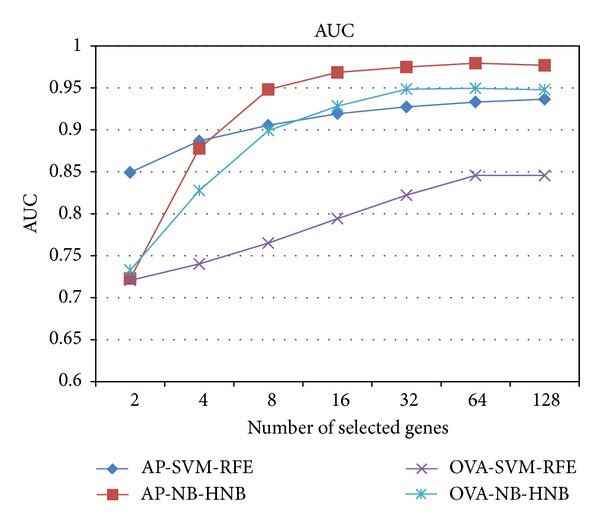
AUC metrics comparing different approaches.

Since the three datasets from KRBDSR are insufficient to demonstrate the performance of our two-step Bayes classification framework, we compared the NB-HNB framework against SVM-RFE using 45 microarray datasets from GEMLeR. The performance results were recorded in terms of (1) classification accuracy, (2) area under the ROC curve (AUC), (3) sensitivity, (4) specificity, and (5) the number of informative biomarkers used for classification. The comparison results of all experiments, including 36 all-possible pairs (AP) datasets and 9 one-tissue-type versus all-other-types (OVA) datasets, are shown in Table 4. In summary, NB-HNB outperformed SVM-RFE on most performance metrics.  Figure 4 presents the average classification accuracy versus the number of selected genes. For all datasets, the accuracy of NB-HNB is better when the number of selected genes is larger than 16. A similar pattern is also observed when comparing AUC between the two approaches (Figure 5). Moreover, the accuracy and AUC do not vary much across different datasets since the standard deviations (Table 4) between NB-HNB and SVM-RFE are similar. 

### 2.3. Experiments on Other Types of -Omics Datasets

We tested whether HNB could also be applied for class prediction from SNP genotyping and SELDI-TOF proteomics datasets. For the bovine dataset, NB-HNB was able to achieve 92% accuracy with 92% sensitivity and as high as 99% specificity using only 33 SNPs, as shown in Table 5. NB-HNB can also be applied to classify cancer proteomics data obtained from SELDI-TOF experiments. For prostate cancer, NB-HNB was able to reach 86% accuracy with 86% sensitivity and 89% specificity using only 8 protein markers. The performance is even better with ovarian cancer, in which NB-HNB demonstrated 98% accuracy at 98% sensitivity and 97% specificity using only 8 protein markers, as shown in Table 6. 

## 3. Conclusions 

The proposed two-step Bayes classification framework outperformed SVM-RFE in all previously reported experiments. Furthermore, we demonstrated that this two-step Bayes classification framework could address the biomarker selection and classification problem beyond the analysis of expression microarray data. Since the two-step Bayes classification framework utilizes Naïve Bayes filtering prior to HNB classification, the complexity of this classification framework is very low permitting analysis of data with many features. 

## 4. Material and Methods

### 4.1. Datasets

The datasets used in the experiments comprise three groups: (1) genomic (2) transcriptomic, and (3) proteomic categories. The first category is SNP genotyping data obtained from the International Bovine HapMap (IBHM) [3] consortium containing 230 individual samples from 19 cattle breeds, each of which has 9,239 SNPs. For the transcriptomic datasets, microarray gene expression data were downloaded from two main repositories: the Gene Expression Machine Learning Repository (GEMLeR) [5] and the Kent Ridge Biomedical Data Set Repository (KRBDSR) [4]. GEMLeR contains microarray data from 9 different tissue types including colon, breast, endometrium, kidney, lung, omentum, ovary, prostate, and uterus. Each microarray sample is classified as tumor or normal. The data from this repository were collated into 36 possible pairings of two tissue types, termed all-possible pairs (AP) datasets and 9 one-tissue-type versus all-other-types (OVA) datasets where the second class is labeled as “other.” All GEMLeR microarray datasets have been analyzed by SVM-RFE, the results of which are available from the same resource. The datasets from KRBDSR contain 7 case-control microarray experiments (tumor versus normal). However, the SVM-RFE results are available only for five datasets from [8, 15, 17], namely, leukemia, colon cancer, breast cancer, lymphoma, and prostate cancer. Ovarian and prostate cancer SELDI-TOF proteomic datasets were obtained from the National Cancer Institute Clinical Proteomics Database (NCICPD) [6]. The information about each dataset, that is, sample size and number of features, is summarized in Table 7. 

### 4.2. Methods

**Figure 6:**
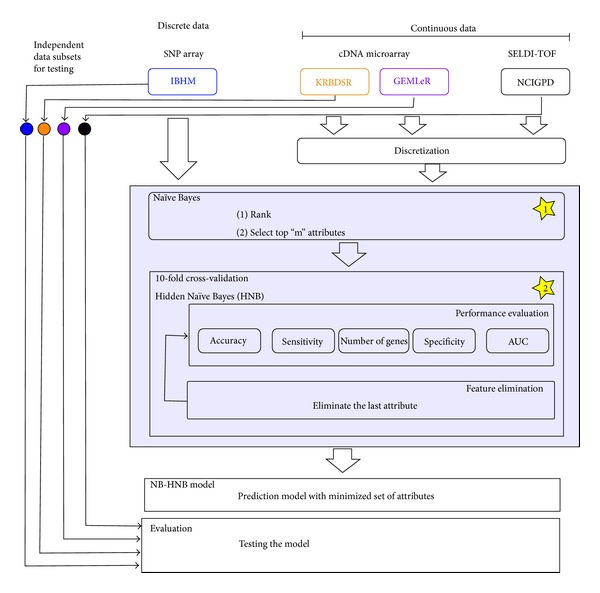
The overall two-step Bayes classification framework.

The two-step Bayes classification framework is composed of two modules: Naïve Bayes (NB) filtering and Hidden Naïve Bayes (HNB) classification.  Figure 6 shows the overall two-step Bayes classification framework. For continuous signal data (e.g., cDNA expression microarray), the data must first be preprocessed by (feature) discretization [20]. The process simply involves processing the data into a series of bins according to the range of values in the dataset. Ten bins were used to group the continuous microarray data by loosely setting each interval (bin) to have the same range. This was done using the Weka discretize module with the following settings: −*B* = 10 and −*M* = −1.0 where −*B* specifies the number of bins and −*M* indicates the weight of instances per interval to create bins of equal interval size. For evaluation of the performance of the NB-HNB model, an independent test dataset is required. We obtained test dataset by randomly selecting 10% of the data from the original dataset that is reserved as a blind dataset (i.e., the data that are analyzed only once using the final classification model) while the rest are used as training data for feature selection and the model classification.

The number of *m* top-ranked features for NB filtering is selected by the user, who inputs the cutoff for individual marker prediction accuracy. From our empirical studies, the top-ranked 40 features provide 75% or greater prediction accuracy. Therefore, we chose this cutoff as the number of markers which can be practically used for HNB processing on a typical desktop computer containing 4 GB RAM with multicore architecture. Obviously with greater computing power, more features could be chosen for higher accuracy. From the NB filtered list of features, an HNB classification model is constructed. The lowest-ranked feature is then removed and another HNB classifier model constructed, which is compared with the previous model for classification accuracy. The process of model building and feature elimination is repeated until the minimum feature subset is obtained which gives a classifier model with the maximum prediction accuracy. 

Intuitively, NB filtering operates by constructing a density estimator using standard Naïve Bayes. The class *c* of sample *E* with attributes can be classified by

(1) $$\begin{array}{c} c\left(E\right)=\arg\underset{c\in C}{\max}\;P\left(c\right)P\left(a_{1},a_{2},\ldots ,a_{n}\;|\;c\right). \end{array}$$

Naïve Bayes assumes that all attributes are independent for a given class. We can then simply represent the above equation by

(2) $$\begin{array}{c} c\left(E\right)=\arg\underset{c\in C}{\max}\;P\left(c\right)\prod_{i=1}^{n}P\left(a_{i}\;|\;c\right). \end{array}$$

The filtering step is performed to quickly extract all the informative features. The ranking is done by sorting the value *P*(*c*)*P*(*a*_*i*_ | *c*), which can be run very quickly by simply counting the number of feature occurrences in each of the corresponding classes; the time complexity is thus *O*(*n*). This, however, does not guarantee that the top-ranked features will contain the optimal set of features that will give the most accurate classification model. The more realistic approach would be to consider all possible dependencies amongst features. However, it has been known that building an optimal Bayesian network classifier is NP hard. To overcome this limitation, we proposed that Hidden Naïve Bayes (HNB) should be used to construct the more realistic classification model from the set of NB filtered attributes.

Instead of building a complete Bayesian graph, which is intractable, HNB is used to construct the dependencies between attributes *A*_*i*_ with a hidden parent *A*_*hp*_*i*__. The modification with the dependency from the hidden parent makes HNB become more realistic by adjusting the weight influenced by all other attributes. A classifier of a sample *E* with attributes [*a*_1_, *a*_2_,…, *a*_*n*_] can be represented by

(3) $$\begin{array}{c} c\left(E\right)=\arg\underset{c\in C}{\max}\;P\left(c\right)\prod_{i=1}^{n}P\left(a_{i}\;|\;a_{hp_{i}},c\right), \end{array}$$

where

(4) $$\begin{array}{c} P\left(a_{i}\;|\;a_{hp_{i}},c\right)=\sum_{j=1,j\neq i}^{n}W_{ij}\times P\left(a_{i}\;|\;a_{j},c\right),\\ W_{ij}=\frac{I_{P}\left(A_{i};A_{j}\;|\;C\right)}{\sum_{j=1,j\neq i}^{n}I_{P}\left(A_{i};A_{j}\;|\;C\right)}, \end{array}$$

where the conditional mutual information *I*_*P*_(*A*_*i*_; *A*_*j*_ | *C*) can be computed as

(5) IP(Ai;Aj ∣ C) =∑ai,aj,cP(ai,aj,c)log⁡⁡(P(ai,aj ∣ c)P(ai ∣ c)P(aj ∣ c)).

## Figures and Tables

**Table 1:** Actual performance results on breast cancer (KRBDSR).

Criterion	Filter	Wrapper methods					Hybrid methods			
	Fisher's ratio	RFE-LNW-GD	RFE-SVM	RFE-LSSVM	RFE-RR	RFE-FLDA	RFE-LNW1	RFE-LNW2	RFE-FSVs-7DK	NB-HNB
Accuracy	0.88	0.78	0.76	0.75	0.74	0.75	0.82	0.88	0.85	0.91
Sensitivity, specificity	0.83, 0.90	0.77, 0.81	0.68, 0.80	0.68, 0.80	0.68, 0.77	0.69, 0.80	0.74, 0.88	0.82, 0.90	0.84, 0.86	0.91, 0.91
Number of genes selected	35	26	33	36	39	28	35	33	21	25

**Table 2:** Actual performance results on leukemia (KRBDSR).

Criterion	Filter	Wrapper methods					Hybrid methods			
	Fisher's ratio	RFE-LNW-GD	RFE-SVM	RFE-LSSVM	RFE-RR	RFE-FLDA	RFE-LNW1	RFE-LNW2	RFE-FSVs-7DK	NB-HNB
Accuracy	0.99	0.99	0.99	0.99	0.48	0.997	0.96	0.99	0.98	1.00
Sensitivity, specificity	0.95, 1.00	1.00, 0.99	0.95, 1.00	0.98, 0.99	1.00, 0.31	0.99, 1.00	0.90, 0.98	0.95, 1.00	0.91, 1.00	1.00, 1.00
Number of genes selected	4	5	4	30	6	5	4	4	3	14

**Table 3:** Actual performance results on colon cancer (KRBDSR).

Criterion	Filter	Wrapper methods					Hybrid methods			
	Fisher's ratio	RFE-LNW-GD	RFE-SVM	RFE-LSSVM	RFE-RR	RFE-FLDA	RFE-LNW1	RFE-LNW2	RFE-FSVs-7DK	NB-HNB
Accuracy	0.90	0.87	0.87	0.91	0.83	0.89	0.91	0.89	0.91	0.93
Sensitivity, specificity	0.92, 0.88	0.89, 0.85	0.92, 0.79	0.97, 0.81	0.77, 0.91	0.93, 0.84	0.93, 0.88	0.93, 0.84	0.93, 0.89	0.93, 0.90
Number of genes selected	16	17	16	22	19	14	10	15	12	23

**Table 4:** Performance comparison between NB-HNB and SVM-RFE on GEMLeR datasets.

Data	NB-HNB		SVM-RFE	
	Accuracy	Number of genes selected	Accuracy	Number of genes selected
AP_Breast_Colon	0.96	22	0.96	8
AP_Breast_Kidney	0.96	17	0.96	8
AP_Breast_Lung	0.94	27	0.94	16
AP_Breast_Omentum	0.95	25	0.96	32
AP_Breast_Ovary	0.96	17	0.96	16
AP_Breast_Prostate	0.99	28	0.99	8
AP_Breast_Uterus	0.96	27	0.95	8
AP_Colon_Kidney	0.97	10	0.98	32
AP_Colon_Lung	0.95	17	0.94	32
AP_Colon_Omentum	0.95	18	0.94	32
AP_Colon_Ovary	0.95	11	0.94	16
AP_Colon_Prostate	0.98	20	0.98	8
AP_Colon_Uterus	0.96	10	0.95	16
AP_Endometrium_Breast	0.97	20	0.97	32
AP_Endometrium_Colon	0.95	21	0.97	32
AP_Endometrium_Kidney	0.98	17	0.98	32
AP_Endometrium_Lung	0.94	27	0.95	32
AP_Endometrium_Omentum	0.92	14	0.9	32
AP_Endometrium_Ovary	0.91	12	0.92	32
AP_Endometrium_Prostate	0.98	20	0.99	4
AP_Endometrium_Uterus	0.9	14	0.76	256
AP_Lung_Kidney	0.96	7	0.96	32
AP_Lung_Uterus	0.93	22	0.93	32
AP_Omentum_Kidney	0.97	18	0.98	16
AP_Omentum_Lung	0.94	24	0.9	128
AP_Omentum_Ovary	0.98	27	0.76	4
AP_Omentum_Prostate	0.98	30	0.98	16
AP_Omentum_Uterus	0.91	15	0.88	16
AP_Ovary_Kidney	0.97	14	0.97	32
AP_Ovary_Lung	0.94	15	0.93	32
AP_Ovary_Uterus	0.88	21	0.89	64
AP_Prostate_Kidney	0.98	20	0.98	2
AP_Prostate_Lung	0.98	14	0.98	4
AP_Prostate_Ovary	0.98	19	0.98	2
AP_Prostate_Uterus	0.97	28	0.99	2
AP_Uterus_Kidney	0.96	12	0.97	32

Average	0.954	18.89	0.94	30.5

Standard deviation	0.02568		0.05357	

OVA_Breast	0.94	15	0.96	32
OVA_Colon	0.96	19	0.97	16
OVA_Endometrium	0.97	6	0.96	2
OVA_Kidney	0.98	20	0.98	8
OVA_Lung	0.97	24	0.97	4
OVA_Omentum	0.95	3	0.95	2
OVA_Ovary	0.92	10	0.93	32
OVA_Prostate	0.99	13	0.997	2
OVA_Uterus	0.97	21	0.93	32

Average	0.96	14.55	0.96	14.44

Standard deviation	0.02147		0.02198	

**Table 5:** Actual performance result on SNPs data (Bovine) from IBHM.

	Accuracy	Sensitivity	Specificity	Number of selected SNP
NB-HNB	0.92	0.92	0.99	33

**Table 6:** Actual performance result of NB-HNB from SELDI-TOF.

	Accuracy	Sensitivity	Specificity	Number of selected genes
Prostate	0.86	0.86	0.89	8
Ovarian	0.98	0.98	0.97	8

**Table 7:** Summary of the information about each dataset, for example, sample sizes, number of attributes.

SNP array		cDNA microarray				SELDI-TOF	
IBHM [3]		KRBDSR [4]		GEMLeR [5]		NCICPD [6]	
Data	Number of SNP (Number of samples)	Data	Number of genes (Number of samples)	Data	Number of genes (Number of samples)	Data	Number of genes (Number of samples)
Bovine	9239 (497)	Leukemia	7129 (72)	Colon	10935 (286)	Ovarian	15154 (253)
		Colon cancer	2000 (62)	Breast	10935 (344)	Prostate	15154 (266)
		Breast cancer	24481 (78)	Endometrium	10935 (61)		
		Lymphoma	4026 (47)	Kidney	10935 (260)		
		Prostate	12600 (102)	Lung	10935 (126)		
		Lung cancer	7129 (96)	Omentum	10935 (77)		
		Nervous	7129 (60)	Ovary	10935 (198)		
				Prostate	10935 (69)		
				Uterus	10935 (124)		
